# Splenic Injury During Percutaneous Nephrolithotomy: A Case Report of a Rare Complication

**DOI:** 10.7759/cureus.6298

**Published:** 2019-12-05

**Authors:** Deniz Noyan Ozlu, Kamil Gokhan Seker, Emre Sam, Feyzi Arda Atar

**Affiliations:** 1 Urology, Bakirkoy Dr. Sadi Konuk Training & Research Hospital, İstanbul, TUR

**Keywords:** percutaneous nephrolithotomy, splenic injury, conservative management

## Abstract

Percutaneous nephrolithotomy has often been the preferred method for large and complex kidney stones. During percutaneous access to the collecting system, we encounter organ injuries due to anatomic neighborhoods. However, splenic injury is a relatively rare complication. We aimed to report how the complication process was managed conservatively in our case with transsplenic access. Then, a brief literature review on management strategy in similar conditions is highlighted.

## Introduction

Percutaneous nephrolithotomy (PNL) has now become the standard surgical method for the treatment of large or complex kidney stones. Although PNL is typically a safe and well-tolerated procedure; as with all surgeries, it has its own complications. Generally, minor complications are observed. Major complications such as injury in neighboring organs, pleural injury, bleeding, or infection are generally related to the percutaneous access to the collecting system [1].

Spleen injury is a rare condition that occurs during PNL. It has some rare samples in the literature as case presentations and many approaches ranging between observation and splenectomy. In this case presentation, our aim was to present a case in which dilatation was applied through the transsplenic approach during PNL and was conservatively treated. 

## Case presentation

**Figure 1:**
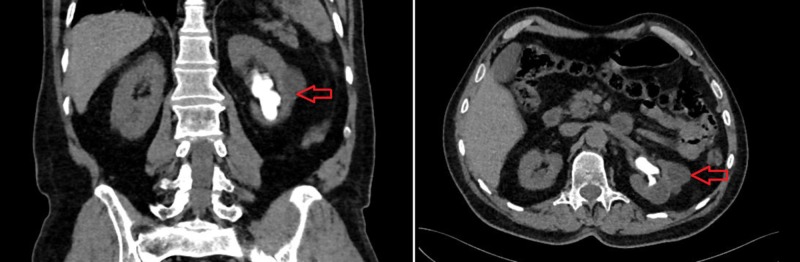
Coronal (left) and axial (right) view of the staghorn calculi filling his left kidney pelvis and lower pole

A 79-year-old male patient was admitted with left side pain. A staghorn calculi filling his left kidney pelvis and lower pole was detected in computed tomography (CT) (Figure 1). The patient was informed about the treatment options, and PNL was planned. Because it is thought that it provides access to more calyx of the kidney and thus increases the stone-free rates, an upper calyceal entry was made between 11th and 12th ribs in the prone position under inspiration. A 14 F nephrostomy tube was inserted after fragmentation of the stones, and PNL was completed without any complications. 

**Figure 2:**
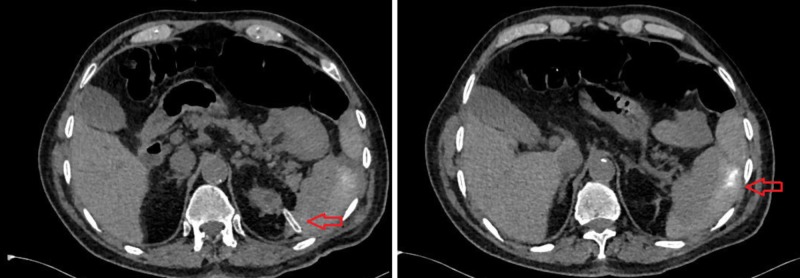
The nephrostomy tube transplenically reached the kidney (left) and the collection area, which extended to 33 mm at its widest in the perisplenic area (right)

**Figure 3:**
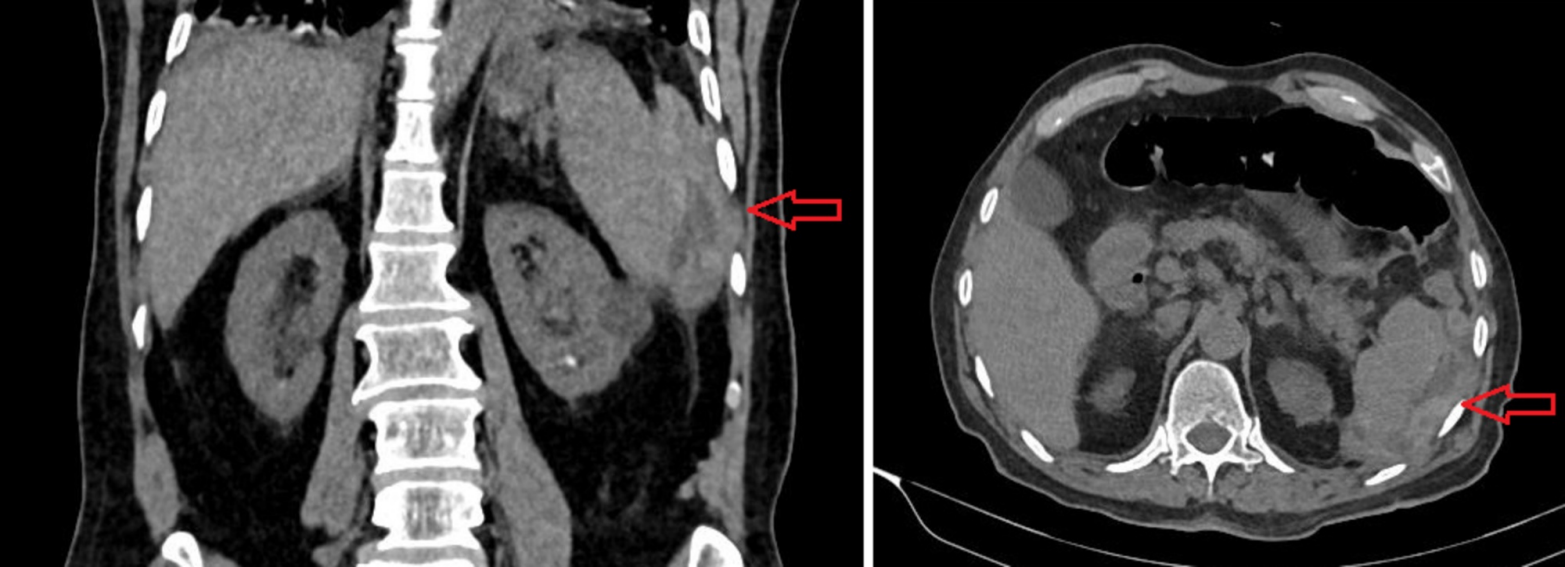
Coronal (left) and axial (right) view of the loculated collection of approximately 111x54 mm in the perisplenic area

Preoperative hemoglobin (Hg) of the patient was 13.6 g/dl, and it was detected as 8.51 g/dl on the first postoperative day. Two units of blood transfusion was performed. A noncontrast CT scan revealed a collection area, which extended to 33 mm at its widest in the perisplenic area and it was observed that the nephrostomy tube transplenically reached the kidney (Figure 2). Since the patient had a stable course during the follow-ups and no decrease in Hg value, the nephrostomy tube was removed on the postoperative fourth day. On follow-up, the patient had left side pain, generalized abdominal tenderness, and a fever of 38.5 degrees. The control CT showed irregularities consistent with a laceration on the middle posterior side of the spleen, and a loculated collection of approximately 111x54 mm in the perisplenic area (Figure 3). No intervention was considered for the patient at this stage. Blood cultures were taken from the patient who had fever under ceftriaxone treatment, and the infectious disease department was consulted. Piperacillin/tazobactam treatment was initiated with the recommendation of the infectious disease department. As the patient's fever continued, the interventional radiology department placed a percutaneous drainage tube in the collection. The result of the aspiration material culture was sterile. The patient continued on bed rest with the drainage tube in situ for two weeks. On the 17th day of his follow-up, the drainage tube was removed and he was discharged as his hemodynamics was stable.

## Discussion

Colon, duodenum, liver, spleen, and pleura are always under injury risk during percutaneous access due to their anatomic neighborhood to the kidney. On the other hand, liver and spleen injury occur rarely and the possibility of spleen injury is rare with subcostal accesses [2]. Gnessin et al. performed postoperative noncontrast CT scans on the patients undergoing PNL. They found the spleen injury ratio at 0.2%. The sensitivity of CT in the diagnosis of postoperative complications after PNL was 92.7% [3].

Although subcostal access is an acceptable approach for renal pelvis, lower, or medial calyx stones, upper calyx access is more suitable for upper ureter, upper calyx, and staghorn calculi. A supracostal approach is inherently related to more complications [4]. Hopper and Yakes performed CT scanning on 43 patients in both the supine and prone positions during inspiration and expiration, and stated that spleen injury risk was minimal when intercostal upper calyx access was performed with complete expiration. They also stated that spleen injury risk increased to 13% through the application of upper calyx access in inspiration and to 33% through 10-11th intercostal access [5].

Other risk factors for spleen injury are splenomegaly and retrorenal spleen [6,7]. Splenomegaly increases the risk of splenic injury by positioning the spleen on the posterolateral aspect of the kidney, and this situation is accepted as a relative contraindication for the left-sided PNL [6]. Retrorenal spleen is an anatomic variant defined by Hopper and Chantelois. They detected retrorenal spleen with a ratio of 21.1% in the supine position and 15.4% in the prone position in a CT review of 73 patients. However, in 7.6% of patients, the retrorenal spleen is at risk of injury during upper calyceal entry below 30 degrees [7].

Currently, preoperative CT scans are routinely used to address patient-specific factors such as retrorenal colon, retrorenal or accessory spleen, or other factors that may increase the risk of injury, and to minimize pleural and intra-abdominal complications. Ng et al. reported that by using a preoperative prone inspiratory-expiratory CT, a percutaneous approach to the kidney can be better calculated, which minimizes potential injuries [8]. Osman et al. asserted that the first percutaneous access should be ultrasound-guided to minimize visceral organ injury risk [9]. Shah et al. suggested that the medial of the posterior axillary line should be used for punction during full inspiration to slide the kidney to the subcostal and then the needle should be inserted during expiration to avoid the spleen and lungs from needle path [6].

In the literature, a relatively low number of spleen injuries were reported during PNL and the use of spleen preserving treatment methods was noticed in most case reports. Splenic injury can be managed conservatively but splenectomy may be required in case of severe hemorrhage. Thus, early diagnosis is important. Spleen injury should immediately be considered in case of hemodynamic instability in the left-sided PNL [2]. Gnessin et al. suggested that CT should be taken in postop 24th hour in the following situations: if a kidney with an anatomic abnormality has been intervened, a preoperative retrorenal colon has been detected, multiple interventions with risk of perinephric hematoma, and in case of transfusion requirement and upper pole entrances with risk of liver or spleen injury [10]. Postoperative CT scan routine may be recommended especially in upper pol approaches to prevent early nephrostomy tube removal in case of injury. But when the low incidence of visceral injury is considered, this scanning method can also be stated to be cost-effective. Routine postoperative CT at upper pole accesses is not an approach we use in our clinical practice. In this case, a postoperative CT scan was performed and splenic injury was detected due to Hg decrease requiring postoperative transfusion. 

Splenectomy following PNL was reported for injuries that are not suitable for conservative treatment due to splenic perforation, ruptured hematoma and excessive bleeding [6,11]. Shah et al. reported two spleen injury cases which required exploratory laparotomy. Splenectomy was performed in one of these patients due to ruptured hematoma. In the other case, fibrin glue was placed on the laceration due to the absence of active bleeding detected intraoperatively [6]. Splenectomy exposes the patient to a significantly higher infection risk compared to the normal population. Polyvalent pneumococcal vaccines are therefore given to patients with splenectomy and the patients should be advised to seek early medical help in the event of a febrile illness.

Conservative treatment includes serial measurements of Hg and hematocrit levels. Selective embolization of the splenic artery has been described in the literature as an alternative to surgery [12]. Thomas et al. performed PNL from the 10th and 11th intercostal spaces in a morbidly obese patient. In the imaging, the nephrostomy tube was in the transsplenic area. Hemostasis was provided through the collagen-thrombin hemostatic agent from a nephrostomy tube [13]. Desai et al. maintained stable hemodynamics without the requirement of exploratory intervention by using Gelfoam pledgets and inserting a urethral stent in a similar case [2]. In our patient, no intervention was performed and he was treated conservatively.

Where damage was discovered before removal of the nephrostomy tube, in some cases the catheter was left in place for 12 to 14 days [14,15]. Schaeffer et al. followed up their patient through hemodynamic and Hg measurements for 72 hours after the removal of the nephrostomy tube [14]. Carey et al. removed the nephrostomy tube after a negative antegrade nephrostogram [15].

In a series of three cases reported by Schaeffer et al. [14] the highest blood loss was observed in the patient whose injury was not noticed until the removal of the nephrostomy tube. In the other two cases, the injuries were incidentally detected without removing the nephrostomy tube and thus long-term drainage and less blood loss were provided. In this respect, the case where the nephrostomy was removed before the injury is similar to the case we presented. Therefore, the long-term nephrostomy tube drainage approach seems to be more accurate in transsplenic inserted catheters. On the other hand, long-term nephrostomy tube drainage has disadvantages such as increased postoperative pain and maintenance of the drainage tube.

## Conclusions

Spleen injury during PNL is a rare complication that may require splenectomy. Early diagnosis and treatment of splenic injuries is important to prevent potential morbidity and mortality. Hemodynamically stable patients can be managed conservatively with bed rest, close monitoring, delayed removal of nephrostomy tubes, and the use of additional hemostatic agents. Splenic preservation should be considered whenever possible to avoid the long-term risks such as infectious complications caused by splenectomy.
